# Supplementary material for: Capabilities of Grazing Incidence X-ray Diffraction in the Investigation of Amorphous Mixed Oxides with Variable Composition
Source: Materials (Basel). 2022 Mar 15;15(6):2144. doi: 10.3390/ma15062144 (PMC8949208; doi:10.3390/ma15062144)
Supplement: Supplementary file 1 [file materials-15-02144-s001.zip › materials-1621242-supplementary.pdf]

Supporting Material

GIXRD and XRR data

Figure S1 shows Grazing Incidence X-Ray Diffraction (GIXRD) analysis carried out on samples composed of pure Ta_2O_5 prepared at decreasing Ar pressures. The absence of Bragg-like peaks and the presence of broad features at low angles ($2\theta < 40°$) is a clear confirmation of the amorphous nature of the deposited oxides. In this case, GIXRD curves are perfectly superimposable as profile features are almost identical.

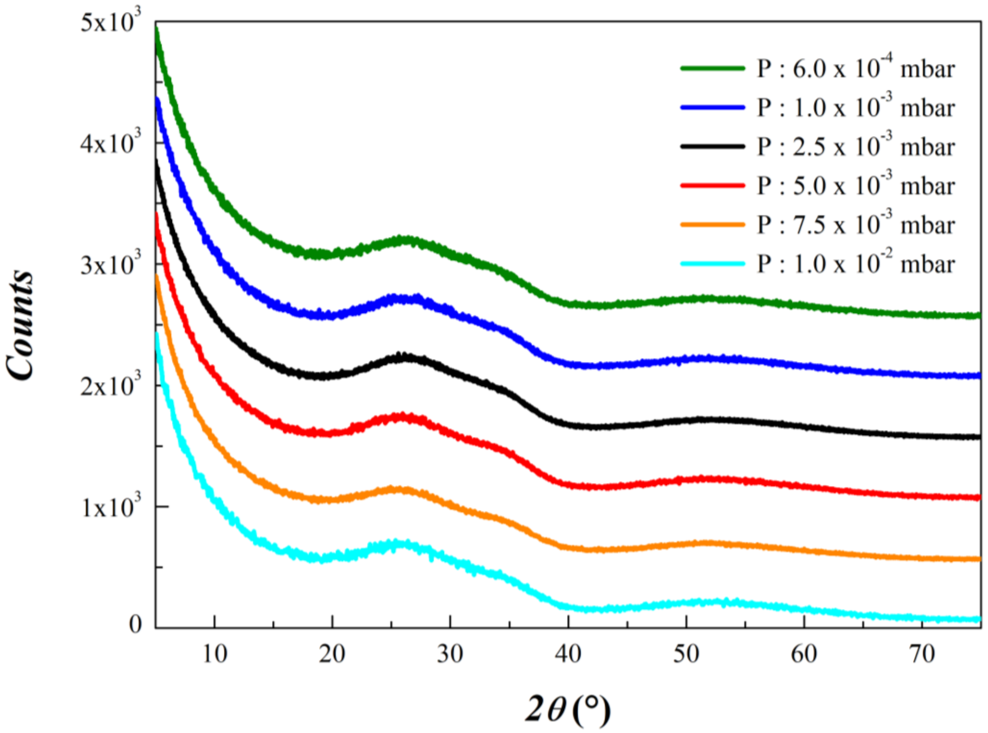

Figure S1. GIXRD diffractograms acquired at fixed incidence angle of 1.3° on the samples obtained at decreasing Ar pressures. A stack was applied to the curves for the sake of a better clarity.

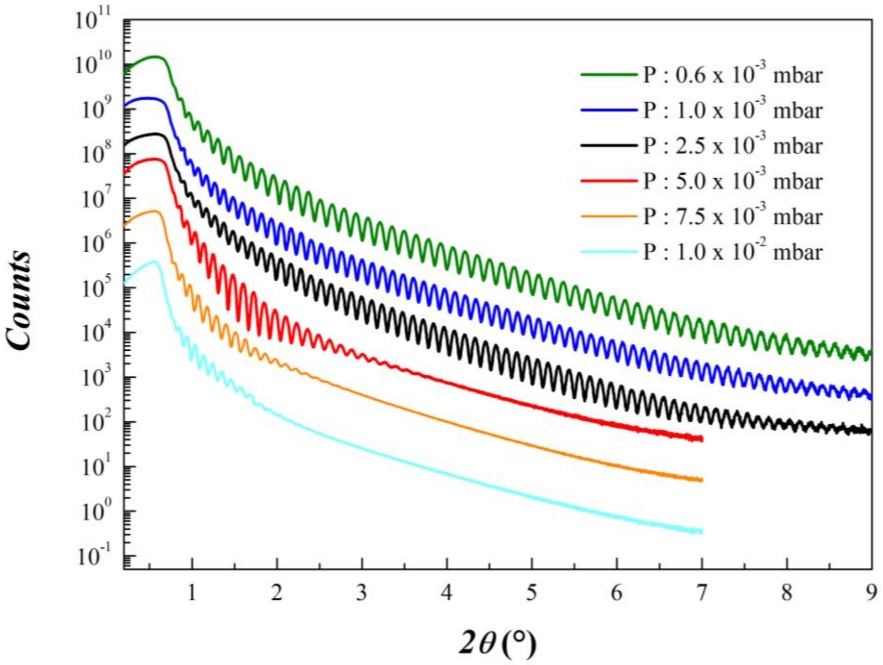

Figure S2. Comparison of XRR curves on Ta_2O_5 samples obtained at decreasing Ar pressures from 1.0 x 10^{-2} mbar to 0.6 x 10^{-3} mbar. For pressures below 2.5 x 10^{-3} mbar the experimental data were recorded in a reduced range because of the smaller extension of interference fringes and therefore of useful data. Ar flow was kept at 3.9 sccm.

Figure S2 shows XRR results referred to the same series of samples. At first sight it is possible to note that the *ω-2θ* curve undergoes important changes by decreasing Ar pressure from 1.0 x 10^{-2} to 0.6 x 10^{-3} mbar. By observing the most important features of XRR curves, it is possible to obtain preliminary qualitative information. (i) From the "step" at low angles, corresponding to the total reflection, is easy to observe that an increase in pressure determines a decrease in density of the deposited oxide. (ii) The frequencies of Kiessig oscillations indicate that sample thicknesses range from 50 to *ca.* 70 nm. (iii) The experimental feature marked by the major variation over the decreasing synthesis pressures is the extent of Kiessig oscillations in *2θ* angle. For the largest working Ar pressure (1.0 x 10^{-2} mbar) oscillations only extend to *ca.* 2.5°, showing a large roughness of the deposited material. A decrease in Ar pressure to 5.0 x 10^{-3} mbar determines an increase in the oscillation range up to *ca.* 3.75°. Then, if Ar pressure is lowered to reach 2.5 x 10^{-3} mbar, we can observe a dramatic change in XRR oscillations range which extends up to 8° in *2θ*. This feature accounts for a better quality and compactness of the samples for working pressures below 5.0 x 10^{-3} mbar. Finally, a further decrease in Ar pressure to 0.6 x 10^{-3} mbar results in further little improvement of sample quality since oscillations are well evident up to *ca.* 9°.
Table S1 illustrates the best XRR fit parameters concerning thickness, density and roughness. Values obtained are in agreement with previously listed qualitative assumptions.

Table S1. Best fit parameters referred to XRR curves illustrated in Figure S2.

Deposition Pressure (10^{-3} mbar /Ar)	Thickness (nm)	Density (g/cm^3)	Roughness (nm)
10	70.3 (5)	6.2 (1)	2.2 (1)
7.5	67.9 (5)	6.7 (1)	2.1 (1)
5.0	64.6 (5)	7.8 (1)	1.7 (1)
2.5	71.3 (5)	7.9 (1)	0.5 (1)
1.0	70.9 (5)	8.0 (1)	0.4 (1)
0.6	60.6 (5)	8.0 (1)	0.4 (1)

Combining GIXRD and XRR results, we can affirm that, in case of pure Ta_2O_5, low angles scattering features remain the same at different pressures even if the density varies. This provides a further confirmation that differences in GIXRD highlighted in the paper can only be ascribed to different oxide ratios in the mixed samples.
Combining GIXRD and XRR results, we can affirm that, in case of pure Ta_2O_5, low angles scattering features remain the same at different pressures even if density varies. This provides a further confirmation that differences in GIXRD highlighted in the paper can only be ascribed to different oxide ratios in the mixed samples.
The fitting of GIXRD diffractograms was carried out by a linear combination of a Lorentzian function (to simulate the background signal) and one or two Gaussian peaks modelling the scattering features. In the main text we have already described the reasons behind the choice of the Gaussians. The following Figure S3 shows the comparison of background best-fits involving different functions: *1/ax* (a), exponential (b) and Lorentzian (c). For the test we considered the GIXRD of pure Ta_2O_5 since it is characterized by the largest intensity. The Lorentzian function leads to the best agreement between theory and experimental data as indicated by the largest R squared value.

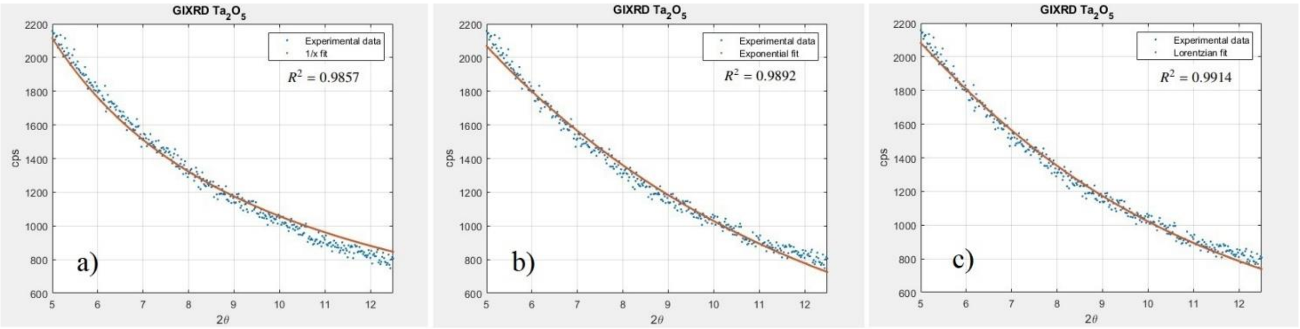

Figure S3. Simulation of GIXRD background in the range 5-12 ° in 2θ with different functions: a) $y = \frac{1}{ax}$ (with a = 9.45 x 10^{-5}), b) $y = ae^{bx}$ (with a = 4159 and b = -0.140) and c) $y = \frac{AB}{\pi x^2 + B^2}$ (with A = 38787 and B = 12.18).

RBS data

Figure S4 reports RBS spectra and best-fit curves for the different samples investigated (apart from those of sample #3 illustrated in the Main Manuscript, Figure1). Figure S4a) and S4d) show the spectra collected for SiO_2 and Ta_2O_5 oxide films and Figure S4b) and S4c) the spectra acquired for the SiO_2-Ta_2O_5 mixed oxide layers. To better enhance the difference between the Si contribution from the substrate and the films, the spectra for the mixed oxide layers had been acquired in glancing configuration.

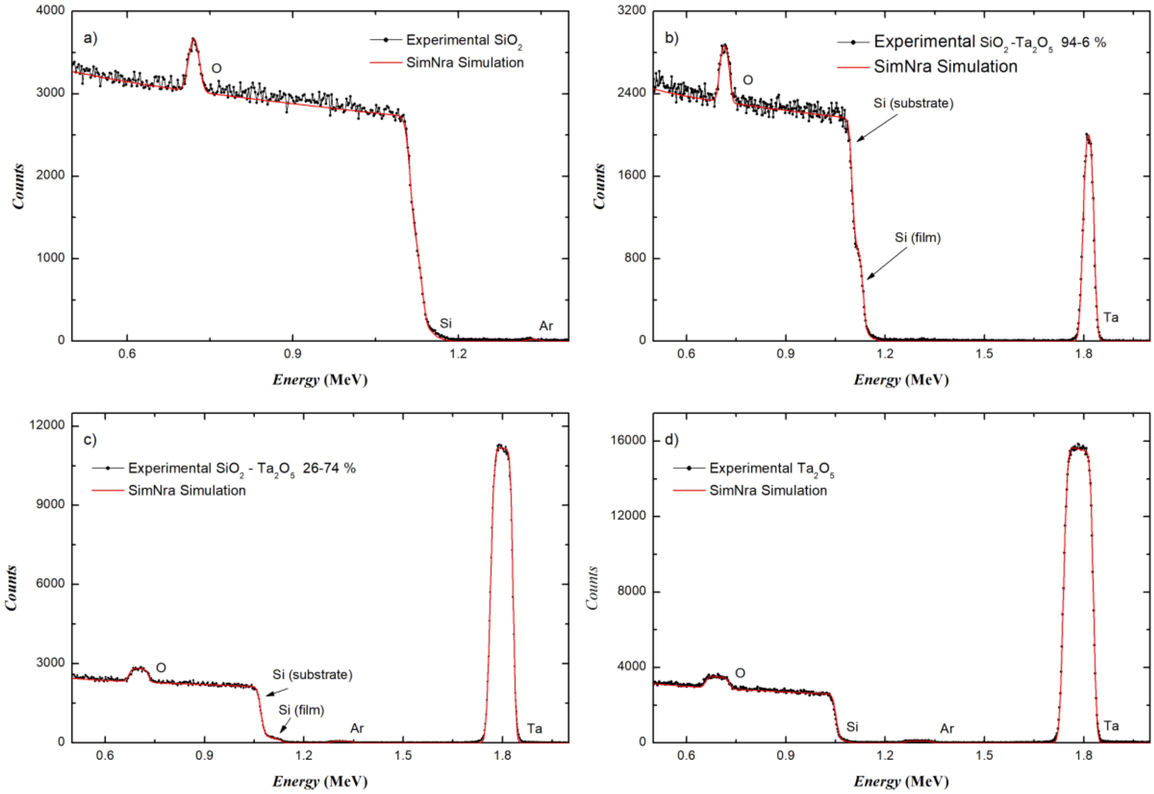

Figure S4. Rutherford Backscattering Spectrometry Experimental Spectra and SimNra curves simulation obtained for S3a) #1, S3b) #2, S3c) #4 and S3d) #5 films.

The atomic percentage of the chemical elements, proportional to the yield of the different signals, has been reported in Table S2 and then converted into a volumetric fraction of SiO_2–Ta_2O_5 to be compared with SE data analysis, as summarized in Table S3. Furthermore, the spectra show evidence of a small

percentage of Ar atomic concentration which increases from 0.5 at% to 2.8 at% along with the content of Ta (a similar effect was found in M. Cevro 1995, Thin Solid Films, 258, 91—103).

Table S2. Atomic Concentration percentage of the mixed oxide films determined by RBS data analysis.

Film	Si (at %)	Ta (at %)	O (at %)	Ar (at %)
#1	31.00	/	68.50	0.50
#2	30.00	2.95	67.05	/
#3	16.00	10.30	72.70	1.00
#4	6.50	19.80	71.70	2.00
#5	/	25.30	71.90	2.80

SE data

Figure S5 reports the pseudo-dielectric function spectra - real and imaginary part of $\tilde{\varepsilon} = \varepsilon_1 + i\,\varepsilon_2$ - of the oxide thin films ($\varepsilon_1 = n^2 - k^2$ and $\varepsilon_2 = 2nk$ with n the refractive index and k the extinction coefficient).

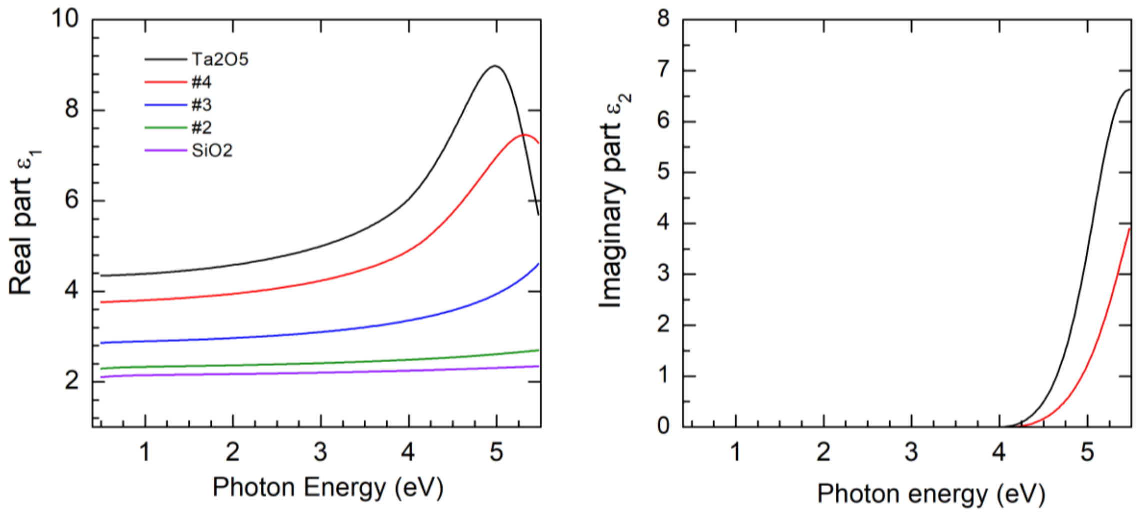

Figure S5. Real (left) and Imaginary (right) part of the complex dielectric function for all the analyzed films, as obtained by ellipsometric spectra analysis. The imaginary part is not reported when negligible.

The spectra are derived (see Main Manuscript) by best-fitting simulated spectra with physical oscillator lineshapes to the ellipsometric experimental ones. In a different approach, these spectra should be analyzed in the so-called Effective Medium Approximation (EMA) as a linear combination of the end-member SiO_2 and Ta_2O_5 dielectric functions, weighted through their volumetric percentages, *i.e.*

$$\tilde{\varepsilon}_{mix} = f_{SiO_2}\,\tilde{\varepsilon}_{SiO_2} + f_{Ta_2O_5}\,\tilde{\varepsilon}_{Ta_2O_5}$$

In Table S3 we report the comparison between the volumetric fractions of the end-member oxides, as evaluated both from RBS data analysis and this EMA approach to SE data. The agreement between the corresponding volumetric fractions is remarkably good, giving evidence of the compositional homogeneity and stoichiometry of these mixed oxide compounds.

Table S3. Comparison of the actual composition of the oxide thin films, as determined by RBS and SE data analyses.

Film	RBS (volumetric fraction %)		SE EMA(volumetric fraction %)	
	SiO_2	Ta_2O_5	SiO_2	Ta_2O_5
# 1	100	/	100	/
# 2	94	6	92.5	7.5
# 3	62	38	60.2	39.8
# 4	26	74	25.4	74.6
# 5	/	100	/	100
